# EXPLORING THE GENERATIVITY OF A MENTORING PROGRAM FOR MEDICAL STUDENTS AND OLDER ADULTS

**DOI:** 10.1093/geroni/igae098.1634

**Published:** 2024-12-31

**Authors:** Leland Waters, Shannon Arnette, Madeline McIntyre, Catherine Tompkins, Sarah Marrs

**Affiliations:** Virginia Commonwealth University, Richmond, Virginia, United States; Virginia Commonwealth University, Richmond, Virginia, United States; Virginia Commonwealth University, Richmond, Virginia, United States; George Mason University, Fairfax, Virginia, United States; Virginia Commonwealth University, Richmond, Virginia, United States

## Abstract

The Virginia Geriatric Education Centers’ Geriatric Workforce Enhancement Program partners with the Virginia Commonwealth University’s School of Medicine to provide a Mentoring Program required for all first-year medical students. Two students are paired with independent, community-dwelling older adult mentors and are required to meet with their mentor three times over the course of two semesters. The students are required to make initial contact and meet the mentor at a time and place of the mentor’s convenience. Mentor volunteer retention was a challenge faced due to pandemic protocols, therefore, limiting the in-person relationships built throughout the program. However, by allowing for both in-person and virtual mentoring experiences, volunteer retention has increased post-pandemic. The students have reading assignments and guided interview questions, which are used as a starting point for conversations with their mentors. The first assignment on aging and health introduces the processes of normal aging, activities of daily living, instrumental activities of daily living, and functional status that is determined by looking at both physical and cognitive actions. The second assignment is a life-space assessment and interview about quality of life. The third assignment focuses on generativity, an evidence-based, psychosocial concept, which is defined as a desire by older adults to nurture and guide upcoming generations, to make meaning of their own lives. This presentation will provide qualitative analysis supporting a generative experience in which the mentors provide their wisdom and the students have an opportunity to develop underlying human relationships that contribute to person-centered care.

